# Phylogeny-Based Comparative Analysis of Venom Proteome Variation in a Clade of Rattlesnakes (*Sistrurus sp.)*

**DOI:** 10.1371/journal.pone.0067220

**Published:** 2013-06-24

**Authors:** H. Lisle Gibbs, Libia Sanz, Michael G. Sovic, Juan J. Calvete

**Affiliations:** 1 Department of Evolution, Ecology and Organismal Biology, Ohio State University, Columbus, Ohio, United States of America; 2 Ohio Biodiversity Conservation Partnership, Ohio State University, Columbus, Ohio, United States of America; 3 Instituto de Biomedicina de Valencia, CSIC, Valencia, Spain; University of Arkansas, United States of America

## Abstract

A long-standing question in evolutionary studies of snake venoms is the extent to which phylogenetic divergence and diet can account for between-species differences in venom composition. Here we apply phylogeny-based comparative methods to address this question. We use data on venom variation generated using proteomic techniques for all members of a small clade of rattlesnakes (*Sistrurus sp*.) and two outgroups for which phylogenetic and diet information is available. We first complete the characterization of venom variation for all members of this clade with a “venomic” analysis of pooled venoms from two members of this genus, *S. milarius streckeri* and *S. m. milarius*. These venoms exhibit the same general classes of proteins as those found in other *Sistrurus* species but differ in their relative abundances of specific protein families. We then test whether there is significant phylogenetic signal in the relative abundances of major venom proteins across species and if diet (measured as percent mammals and lizards among all prey consumed) covaries with venom composition after phylogenetic divergence is accounted for. We found no evidence for significant phylogenetic signal in venom variation: K values for seven snake venom proteins and two composite venom variables [PC 1 and 2]) were all nonsignificant and lower (mean = 0.11+0.06 sd) than mean K values (>0.35) previously reported for a wide range of morphological, life history, physiological and behavioral traits from other species. Finally, analyses based on Phylogenetic Generalized Least Squares (PGLS) methods reveal that variation in abundance of some venom proteins, most strongly CRISP is significantly related to snake diet. Our results demonstrate that venom variation in these snakes is evolutionarily a highly labile trait even among very closely-related taxa and that natural selection acting through diet variation may play a role in molding the relative abundance of specific venom proteins.

## Introduction

Snake venom proteins are one of the most widely studied types of animal toxins (for general reviews see [1], [2]). These proteins largely belong to a few major protein families, including enzymes (serine proteinases, Zn^2+^-metalloproteinases, L-amino acid oxidase, group II PLA_2_s) and proteins without enzymatic activity (disintegrins, C-type lectin-like proteins, vasoactive peptides, myotoxins, CRISP, nerve and vascular endothelium growth factors, cystatin and Kunitz-type protease inhibitors) [3]–[8]. They function by interfering with the coagulation cascade, haemostatic system, and tissue repair acting to immobilize, kill and digest the prey of venomous snakes (for recent review see [9]).

Variation in venom composition at different biological levels is widespread [10], [11] and has been argued to be an adaptation that has evolved via natural selection, allowing different species to capture and digest different prey [12]–[14]. However, others have proposed that, due to the high toxicity and large doses of venom that are injected, variation in venom composition is unlikely to be subject to natural selection for lethal effects on prey, and that much of the variation in venom proteins may be a by-product of neutral evolutionary processes [15]–[17]. Under this hypothesis, interspecific differences in venom composition should be closely related to the degree of phylogenetic divergence between species.

One rarely-used approach to study the evolutionary basis of venom variation is to examine patterns of interspecific variation in venom in the context of phylogenetic relationships among a group of related species [12], [14], [18]. Specifically, phylogeny-based comparative methods [19], [20] offer ways to address key questions about venom differentiation between species. One long-standing question is the degree to which venom variation covaries with levels of phylogentic divergence between species. One way to measure such a relationship is to determine the level of phylogenetic signal in venom composition across a set of species for which a phylogeny has been established. Phylogenetic signal refers a tendency (pattern) for evolutionarily related organisms to resemble each other, with no implication as to the mechanism that might cause such resemblance (process) (20). Moderate levels of signal would support the hypothesis that neutral evolutionary mechanisms such as genetic drift are responsible for variation in venom composition across a set of related species whereas low or exceptionally high levels of signal would leave open a role for selection acting on venom composition through diet variation. If this is the case, evidence for selection could be examinedusing other comparative methods (19) to see if correlations exist between venom composition and measures of diet after taking expected similarity due to phylogeny alone into account. Here, we explore these questions using data from high resolution proteomics-based analysis of venom composition combined with phylogenetic and diet information for all members of a small clade of rattlesnakes (*Sistrurus sp.*).

**Figure 1:**
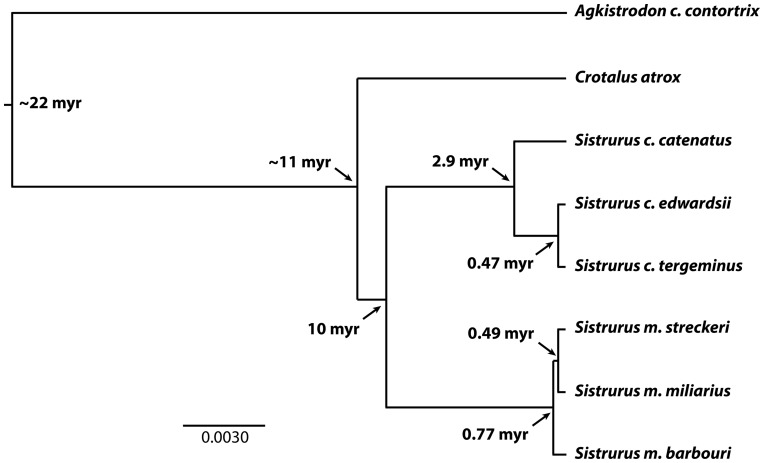
Species tree based on multi-locus data showing phylogenetic relationships and approximate dates of divergence for all *Sistrurus* taxa [22] and *Crotalus atrox* and *Agkistrodon c. contortrix* outgroups [57]. Branch lengths for the in group taxa and *Agkistrodon* are as in Kubatko *et al*. [22]. Branch lengths for *C. atro*x were estimated based on mtDNA ATP6–8 gene sequence divergences between *C. atrox* [30] and *S. miliarius* and *S. catenatus* [22].

*Sistrurus* rattlesnakes are a genus of New World pitvipers (Viperidae; Crotalinae) consisting of two named species: massasauga (*S. catenatus*) and pigmy (*S. miliarius*) rattlesnakes. Each species consists of three named subspecies (*S. c. catenatus*; *S. c. tergeminus*, and *S. c. edwardsii*, and *S. m. milarius*, *S. m. barbouri*, and *S. m. streckeri* ) [21]. Recently, Kubatko et al. [22] conducted a phylogenetic analysis of the genus based on multilocus data in which they generated a species tree with branch lengths based on multiple gene trees, estimated dates of divergence, and conducted tests of the genetic distinctiveness of each subspecies (Figure 1). These tests showed evidence for genetic distinctiveness of all subspecies. Further, all taxa surveyed to date show substantial differences in venom composition [23]. This is a key trait involved in prey capture and digestion and differences have functional consequences in terms of the ability to subdue ecologically-diverse prey [24]. In particular, toxicity to mammals appears to be a key axis along which venom function has evolved with whole venom from taxa showing unusually high (*S. c. catenatus*) and unusually low (*S. m. barbouri*) LD_50_ values for mice [24]. These data suggest that the proportion of mammals in the diets of different *Sistrurus* may be related to venom composition and imply a role for natural selection in generating differences in venom composition between taxa. However, the extent that levels of phylogenetic divergence alone could account for interspecific differences in venom is unknown.

Here, we examine the association between phylogenetic divergence, diet and venom variation for *Sistrurus* rattlesnakes. We complete the proteomic-based analysis of the abundances of specific venom proteins (“venomics”) for all *Sistrurus* by analyzing the venom of the two as yet unanalyzed taxa (*S. m. milarius*, Carolina pigmy rattlesnake, and *S. m. streckeri*, Western pigmy rattlesnake). We combine this information with published data on venom composition for other *Sistrurus* [23] and close (*Crotalus atrox*) [25] and distant (*Agkistrodon c. contortrix*) (J Calvete et al., unpublished data) outgroups. We analyze these data in the context of a phylogeny for the group [22] to determine if there is a phylogenetic signal in shifts in venom composition. We then test whether correlations exist between venom composition and diet after taking expected similarity due to phylogenetic divergence alone into account. These analysis complement our previous work on venom variation in *Sistrurus* that has focused on patterns of intraspecific variation in *S. c. catenatus* [26] and the function of venom variation in terms of toxicity to different prey [24].

## Materials and Methods

### Ethics Statement

Permits for sampling snakes were obtained from the St. Louis Zoo (Permit BM 2010-06) issued by M. Duncan and from the Carolina Sandhills National Wildlife Refuge (Special Use Permit issued by L. Housh). The protocol for the collection of venom samples from individual snakes was approved by the Institutional Animal Use and Care Committee of The Ohio State University (Permit Number: 2008A0087). All efforts were made to minimize suffering of the animals while samples were being collected.

### Samples of *S. m. milarius* and *S. m. streckeri* Venom

For proteomic analyses of venom, we obtained venom samples from two sources: captive adult *S. m. streckeri* (n = 5) held at the St. Louis Zoo which had originally been collected from the wild in SE Missouri (Barry County), and wild-caught adult *S. m. milarius* (n = 5) from Carolina Sandhills National Wildlife Refuge in Chesterfield County, South Carolina. Snakes were restrained in clear plastic tubes and then induced to bite the top of a glass beaker that had been covered with Parafilm. Secreted venom was immediately pipetted into a cryovial and stored in liquid nitrogen.

### Isolation and Proteomic Characterization of Venom Proteins

For each taxon, separate pooled samples consisting of equal amounts of venom from five individuals were subject to proteomics analysis as described by Sanz *et al*. [23]. Pooled samples were used because they yield more interpretable HPLC profiles of venom composition that reflect the full range of variation of observed across multiple individual samples (27). Briefly, soluble proteins from 2 mg of crude venom were were separated on a Teknokroma Europa C_18_ (0.4 cm×25 cm, 5 mm particle size, 300 Å pore size) column using an Agilent LC 1100 High Pressure Gradient System equipped with DAD detector and micro-Auto-sampler. The flow-rate was set to 1 ml/min and the column was developed with a linear gradient of 0.1% TFA in water (solution A) and acetonitrile (solution B), isocratically (5% B) for 10 min, followed by 5–25% B for 20 min, 25–45% B for 120 min, and 45–70% for 20 min. Protein detection was at 215 nm and peaks were collected manually and dried in a Speed-Vac (Savant). Reverse-phase HPLC runs were consistently superimposable through their X-axes and the acetonitrile gradient profile, and thus all chromatograms of venom samples from different specimens from the same taxon were directly comparable. Given that the wavelength of absorbance for a peptide bond is 190–230 nm, protein detection at 215 nm allows to estimate the relative abundances (expressed as percentage of the total venom proteins) of the different protein families from the relation of the sum of the areas of the reverse-phase chromatographic peaks containing proteins from the same family to the total area of venom protein peaks in the reverse-phase chromatogram. In a strict sense, and according to the Lambert-Beer law, the calculated relative amounts correspond to the “% of total peptide bonds in the sample”, which is a good estimate of the % by weight (gr/100 gr) of a particular venom component.

Initially, the HPLC-isolated proteins were subjected to N-terminal sequence analysis (using a Procise instrument from Applied Biosystems (Foster City, CA, USA) following the manufacturer’s instructions). Amino acid sequence similarity searches were performed against the available databanks using the BLAST program [28] implemented in the WU-BLAST2 search engine at http://www.bork.embl-heidelberg.de. The molecular masses of the purified proteins were determined by SDS-PAGE (on 12–15% polyacrylamide gels) and by MALDI-TOF mass spectrometry using an Applied Biosystems Voyager-DE Pro mass spectrometer operated in linear mode.

Protein bands of interest were excised from Coomassie Brilliant Blue-stained SDS-PAGEs and subjected to automated reduction with DTT, alkylation with iodoacetamide, and proteolytic digestion with sequencing grade bovine pancreas trypsin (Roche). The tryptic peptide mixtures were dried, redissolved in 70% acetonitrile and 0.1% TFA and 0.65 ul of the digests spotted onto a MALDI-TOF sample holder and analyzed with an Applied Biosystems Voyager-DE Pro MALDI-TOF mass spectrometer, operated in delayed extraction and reflectror modes. For peptide sequencing, the protein digest mixture was loaded in a nanospray capilary and subjected to electrospray ionization mass spectrometric analysis using a QTRap 2000™ mass spectrometer (Applied Biosystems) equipped with a nanospray source (Proxeon Biosystems). Monoisotopic doubly- or triply-charged precursor ions were selected (within a window of ±0.5 m/z) and sequenced by CID-MS/MS using the Enhanced Product Ion mode with Q_0_ trapping option; Q1 was operated at unit resolution, the Q1-to-Q2 collision energy was set to 30 (for m/z ≤700) or 35 eV (for m/z >700), the Q3 entry barrier was 8 V, the LIT (linear ion trap) Q3 fill time was 250 ms, and the scan rate in Q3 was 1000 amu/s. CID spectra were interpreted manually (i.e. *de novo* sequenced) or using the on-line form of the MASCOT program at http://www.matrixscience.com against the SwissProt and NCBInr databases, and against a private database comprising previous assignments of *Sistrurus* venom proteins [23]. MS/MS mass tolerance was set to ±0.6 Da. Carbamidomethyl cysteine and oxidation of methionine were fixed and variable modifications, respectively.

### Phylogeny-based Comparative Analyses

To analyze changes in venom in a phylogenetic context we used data on venom composition and phylogenetic relationships for all *Sistrurus* taxa and close (*C. atrox*) and distant (*Agkistrodon c. contortix*) outgroups. We chose *C. atrox* and *A. c. contortix* as representative outgroups because they are species from either the sister clade to *Sistrurus* (*C. atrox*) or the sister clade to all rattlesnakes (*A. c. contortrix*) [29] and because both species have haemorrhagic venoms which are the same general type of venom found in all *Sistrurus* [18 and JJ Calvete, unpublished data], making them good representatives of ancestral venom composition. We used the species tree topology and associated multilocus-based branch lengths from Kubatko *et al.* [22] as our estimate of the phylogeny for this group with the exception that we added *C. atrox* to the phylogeny. To estimate a value for the multilocus branch length connecting *C. atrox* with the *Sistrurus* ingroup clade, we used divergence estimates based on mtDNA ATP6–8 gene sequences from *C. atrox* [30] and *S. miliarius* and *S. catenatus* [22]. Venom composition for each taxa was measured as the relative percent of total venom composition made up by proteins from distinct families using data from this study and Sanz et al. [23] for *Sistrurus*, Calvete et al. [25] for *C. atrox* and Calvete et al. [unpublished data – see Supplemental Information] for *A. c. contortrix*. For the statstical analysis, percent values for individual proteins for each taxon were arcsin square root transformed to normalize the data and reduce the dependancy between individual values for a given taxon. We also analyzed composite variables in the form of Principle Components 1 and 2 that summarized the variation present across all venom proteins. These were calculated using the Princom subroutine in R [31].

For broad-scale estimates of diet, we used published information based on gut content analyses to estimate the percentage of each taxon’s diet made up of mammals and lizards. Diet data for six of the taxa came from studies with large samples from multiple geographic locations (*S. c. catenatus* [n = 139 samples]; *S. c. tergeminus* [n = 111]; *S. c. edwardsii* [n = 163] (data from 32); *S. m. barbouri* [n = 103] (data from T. Farrell and P. May, unpublished data); *C. atrox* [n = 205](data from 33)) and *A. c. contortrix* [n = 135] (data from 34) whereas samples for the two other taxa were more limited (*S. m. streckeri* [n = 14] (data from 35); *S. m. miliarius* [n = 12] (data from 36) which could compromise accuracy of our diet estimates for these taxa. We chose these broad categories because they represent general differences in diet that could be readily estimated from the available data that may reflect functional differences in venom toxicity among these snakes (see 24). However, we acknowledge that other prey could be important and/or that more sophisticated analyses that integrate across types of prey would be informative if better estimates of diet were available for the taxa with limited samples. Because of the high correlation between percent mammals and lizards in snake diets (see below) we also estimated a composite variable in the form of Principle Component 1 calculated as above.

We used the subroutine multiPhylosignal in the R-package Picante [37] to determine if there was evidence for phylogenetic signal in both venom and diet composition across taxa. This program calculates a K value for each trait which is an estimate of the amount of phylogenetic signal present in the trait being analyzed [20]. As described in Blomberg et al. [20] K is estimated under a generalized least squares modeling approach and is based on the calculation of mean square error of trait variation relative to its expectation under a Brownian Motion model of evolution. A Brownian Motion model assumes a linear relationship between phylogenetic distance and between taxon divergence in traits and is commonly used in population genetics to describe the evolution of characters undergoing random genetic drift. K can vary from 0 (no correspondence between phylogeny and trait variation) to 1 (evolution by Brownian motion, wherein trait differences are correlated with amount of phylogenetic divergence), to greater than 1 (closely related species have diverged in phenotype less than expected based on the levels of divergence). A statistical test of whether the observed K is greater than random expectation was then carried out by randomly assigning trait values to tree tips and calculating a “random” K value 1000 times and comparing the observed value to the distribution of random K values.

Finally, we used the subroutine PGLS in the R-package Caper [38] to examine associations between venom composition and diet independent of similarity due to phylogeny. This method implements Generalized Least Squares models which account for phylogeny by incorporating estimates of relatedness between taxa into comparisons that determine whether an independent trait (here diet) predicts values of another dependent trait (venom composition) (see ref. 19, Chapter 7 for review). It provides a more general and flexible approach to the widely-used independent contrasts methods pioneered by Felsenstein [39]for assessing correlations between traits independent of phylogenetic divergence. In our analyses we assessed whether a measure of diet (PC 1 score based on the abundance of mammals and reptiles in the diet) was significantly associated with the transformed proportions of specific venom proteins and PC 1 and 2 scores. Significance of the association was assessed using a t-test to evaluate whether the slope was significantly different from zero. The critical p-value was adjusted for the number of comparisons made using the Benjamini and Yekutieli False Discovery Rate adjustment described in [40].

## Results

### Proteomic Analysis of *S. m. streckerii* and *S. m. milarius* Venoms

**Figure 2:**
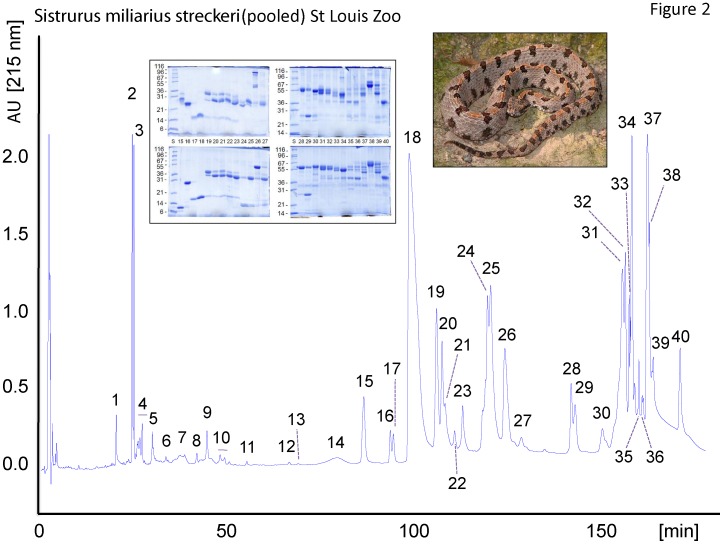
The venom proteome of *S. m. streckeri.* The proteins from 2 mg of pooled crude venom were fractionated on a C_18_ column as described in the Experimental section. HPLC fractions were collected manually and analyzed by SDS-PAGE (insert; under non-reduced (upper panel) and reducing (lower paner) conditions), N-terminal sequencing, and molecular mass determination by ESI-MS or SDS-PAGE. Protein bands excised from SDS-polyacrylamide gel were identified by tryptic peptide mass fingerprinting and CID-MS/MS. The results are listed in Table 1.

**Figure 3:**
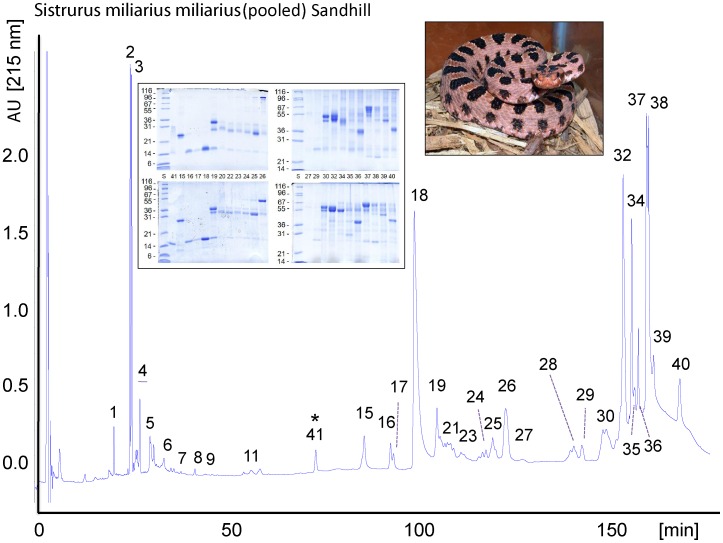
The venom proteome of *S. m. miliarius*. The proteins from 2 mg of crude venom were fractionated on a C_18_ column as described in as described in the Experimental section. HPLC fractions were collected manually and analyzed as in Fig. 2. The results are listed in Table 1.

**Figure 4:**
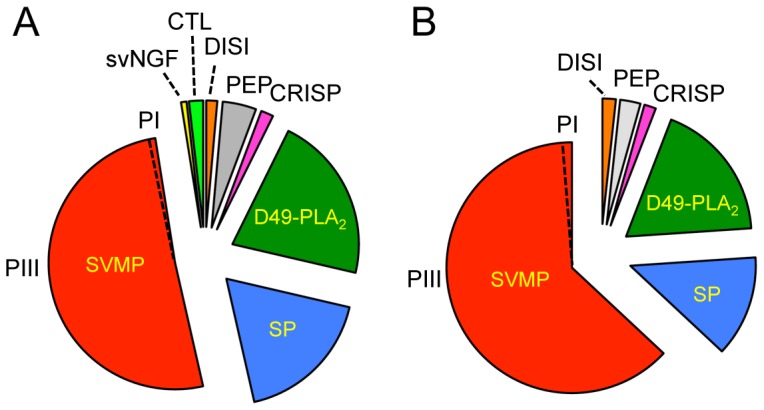
Comparison of the overall venom proteomes of *S.* *m. streckeri* and *S. m. miliarius*. Pie charts display the relative occurrence of proteins from different toxin families in the venoms of *S. m. streckeri* (panel A) and *S. m. miliarius* (panel B). svNGF, snake venom nerve growth factor; CTL, C-type lectin-like protein; DISI, disintegrin; PEP, peptides (including tripeptide SVMP inhibitors, bradykinin-potentiating peptides (BPP), and C-natriuretic peptide, C-NP); CRISP, cysteine-rich secretory protein; D49-PLA2, D49-phospholipase A2; SP, serine proteinase; PI- and PIII-SVMP, snake venom Zn2+-metalloproteinase of class I and class III, respectively. The percentages of the different toxin families in *S. m. streckeri* and *S. m. miliarius* venoms are listed in Table 2.

The venom proteins of *S. m. streckeri* and *S. m. miliarius* were initially separated by reverse-phase HPLC (Figs. 2 and 3). The venoms of these two subspecies yielded superimposable elution profiles and the proteins recovered in apparently equivalent chromatographic peaks were almost indistinguishable by SDS-PAGE (inserts of Figs. 2 and 3) and venomic analyses (Table 1). Only a few protein peaks differentiated the venom proteomes of the Western pigmy rattlesnake, *S. m. streckeri*, and the Carolina pigmy rattlesnake *S. m. miliarius*, eg. serine proteinase Sms-20, PI-SVMP Sms-31, CTL Sms-24, NGF Smm-14 and D49-PLA_2_ Smm-41 (Table 1). In addition to these qualitative differences, the venom proteomes of *S. m. streckeri* and *S. m. miliarius* also exhibit quantitative departures, most notably in their PIII-SVMP content (Table 2; Fig. 4). Notwithstanding the mentioned qualitative and quantitative differences, the Western and Carolina pigmy rattlesnakes share about 95% of their venom proteomes, which comprise 3 major toxin groups (PIII-SVMP> D49-PLA_2_> serine proteinase) and 6 (*S. m. streckeri*) or 4 (*S. m. miliarius*) minor (<5% of the total venom proteins) peptide/protein classes (disintegrin, C-NP, svNGF, CRISP, C-type lectin-like, PI-SVMP).

**Table 1:** Assignment of the reverse-phase fractions from the venoms of *Sistrurus miliarius streckeri* (Sms) and *S. m. miliarius* (Smm), isolated respectively as in Figs.1 and 2, to protein families by N-terminal Edman sequencing, mass spectrometry, and collision-induced fragmentation by nESI-MS/MS of selected peptide ions from in-gel digested protein bands separated by SDS-PAGE (inserts in Figs. 1 and 2).

HPLC Fraction		N-terminal sequence	Molecular mass	Peptide Ion		MS/MS-derived sequence	Protein family
				m/z	z		
**Sms-**	**Smm-**						
1	1	Blocked		624.4	1	ZXPQR	Unknown
2–3	2–3	Blocked		430.1	1	ZNW	SVMPi [∼P01021]
				444.4	1	ZBW	SVMPi [∼P01021]
4	4	EAGEECDCGSPENPCCDAAT	7639				Disintegrin [P22827] (1–73)
		GEECDCGSPENPCCDAAT	7432				Disintegrin [P22827] (3–72)
		EECDCGSPENPCCDAATCK	7375				Disintegrin [P22827] (4–72)
5	5	Blocked		569.8	2	ZNWPAPHIPP	BPP
9	9	GSGCFGLKLDRIGSMSGLGC	1955.8				C-NP [B0VXV8] 182–201
14		Blocked	16^♦^ kDa	556.3	2	NPNPVPTGCR	Nerve growth factor [∼BAG16511]
				682.8	2	AXTMEGNQASWR	
15	15	SVDFDSESPRKPEIQ	24779	569.8	2	SVDFDSESPR	CRISP [∼B0VXV6]
				533.8	2	IVDLHSLR	
16	16	HLIQFETLIMKIAGR	13997	686.8	2	CCFVHDCCYGK	D49-PLA_2_ [ABY77922]
				490.3	2	QICECDR	
				793.2	2	DNIPTYDDKWR	
				753.8	2	HLIQFETLIMK	
19	19	VIGGDECNINEHRFL	36^▪^/38^♦^ kDa	553.2	2	FLVALYHSR	Serine proteinase
				756.7	2	VIGGDECNENIHR	
				885.8	2	ILCAGVLEGGIDTCHR	
				2562.6	1	TFLCGGTLLNEEWVLTAAHCDR	
		HTLIQFETLIMKIAGR	16^▪^/^♦^ kDa				D49-PLA_2_ [ABY77929]
20	20	VIGGDECNINEHR	28^▪^/34^♦^ kDa	553.2	2	FLVALYHSR	Serine proteinase
				756.7	2	VIGGDECNINEHR	
22–23	22–23	(V/I)(I/V)GGDECNINEHR(F/S)L	28^▪^/34^♦^ kDa	553.2	2	FLVALYHSR	Serine proteinase
				756.7	2	VIGGDECNINEHR	
				749.7	2	VVGGDECNINEHR	
				763.3	2	IIGGDECNINEHR	
				885.8	2	ILCAGVLEGGIDTCHR	
				561.6	2	AAYSSLPATSR	
24	24	VVGGDECNINEHRFL	28^▪^/34^♦^ kDa	749.7	2	VVGGDECNINEHR	Serine proteinase
				594.7	2	TVPNEDEQTR	
				559.6	2	TLCAGILEGGK	
24		DCPSGWSYYEGHCYK^*^	26^▪^/13–14^♦^ kDa				C-type lectin-like
25	25	V(V/I)GGDECNINEHRFL	28^▪^/33^♦^ kDa	749.7	2	VVGGDECNINEHR	Serine proteinase
				756.7	2	VIGGDECNINEHR	
				594.7	2	TVPNEDEQTR	
26	26	Blocked	96^▪^/48^♦^ kDa	606.8	2	SAECIDSFQR	PIII-SVMP [B0VXU9]
				861.6	2	MYDTVNVITPIYHR	
				617.9	2	MNIHVALVGLEIWSNR	
				707.8	2	DDCDMADVCTGR	
				678.3	2	LYCFPNSPETK	
				604.8	3	QGAQCAEGLCCDQCR	
27	27	VIGGDECNINEHRFL	28^▪^/33^♦^ kDa				Serine proteinase
28	28	Blocked	54^▪^/56^♦^ kDa	777.3	2	VCSNGHCVDVATAY	PIII-SVMP
				873.8	2	VAVTMTHEXGHNXGXR	
				670.8	3	XYEXVNTMNEXYXPXNXR	
29	29	ALNPEEQRYVELFIV	26^▪^/^♦^ kDa	631.2	3	TWVHEXVNTXNVFYR	PI-SVMP
30		Blocked	54^▪^/56^♦^ kDa	774.6	2	FALVGLEIWSNGDKI	PIII-SVMP [∼B0VXU6]
				587.7	2	IFPCAPQDVK	
			48^▪^/52^♦^ kDa	664.3	2	YVEXVXXWHR	PIII-SVMP
				603.3	2	FTSAGNVC(gh)R	
31, 32	32	NLTPEQQAYLDAKKY	52–54^▪^/^♦^ kDa				PIII-SVMP [∼ABG26978]
31		ND	48^▪^ kDa	736.8	3	IYEIVNTMNEIYIPLNIR	PIII-SVMP [∼B0VXU4]
				600.3	3	YVEFVVVLDHGMYTK	
				875.7	2	LSHQPSTQFSDCSEK	
				534.6	2	GLCCDQCR	
33	33	ALNPEEQRYVELVVV	48^▪^/52^♦^ kDa	526.6	2	GNYYGYCR	PIII-SVMP
35	35	ND	39^▪^/^♦^ kDa	657.8	2	YXEXVVVADHR	PIII-SVMP
37–39	37–39	Heterogeneous	66^▪^/^♦^ kDa	583.1	2	IGNYYGYCR	PIII-SVMP [∼B0VXU5]
				677.2	2	YIELVIVADYR	
				638.1	3	TWVYEIVNTLNEIYR	
				514.6	2	IPCAPEDVK	
				614.4	3	MCGVTQNWESYEPIK	
				2529.1	1	LYCSYNFNGNQIPCVPYYTR	
			23^▪^/^♦^ kDa	547.8	2	YNSNXNTXR	PI-SVMP
				672.8	2	YXEXVVVTDHR	
				631.2	3	TWVHEXVNTXNVFYR	
39	39	ND	48^▪^ kDa	615.2	2	XPDSEAHAVYK	PIII-SVMP [∼B0VXU5]
				717.1	2	XQGEMYXXEPXK	
39	39	AXIYQRYIELVVVAD	39^▪^/^♦^ kDa	657.8	2	YXEXVVVADHR	PIII-SVMP
				912.8	2	VTXSADDTXQAFAEWR	
			26^▪^ kDa	657.8	2	YXEXVVVADHR	PI-SVMP
41		SLLQFNKMIKIMTKK	14026	490.7	2	QICECDR	D49-PLA_2_ [∼ABY77926]
				581.6	2	TDIYSYSWK	
				747.8	2	SGVITCGEGTPCEK	
				770.6	2	AAAVCFGENLPTYK	
				689.9	3	NAIPSYTSYGCYCGWGGR	
				789.3	2	CCFVHDCCYEK	

Apparently identical proteins in Sms and Smm venoms are labelled with the same number. In MS/MS-derived sequences, X = Ile or Leu; Z, pyrrolidone carboxylic acid; B, Gln or Lys. Unless other stated, for MS/MS analyses, cysteine residues were carbamidomethylated; Molecular masses of native proteins were determined by electrospray-ionization (±0.02%) or MALDI-TOF (±0.2%) mass spectrometry. Apparent molecular masses were determined by SDS-PAGE of non-reduced (▪) and beta-mercapethanol-reduced (♦) samples. np, non-peptidic material found; ND, not determined.

**Table 2:** Overview of the relative occurrence of proteins of different families (in percentage of the total HPLC-separated proteins) in the venoms of *Sistrurus m. streckeri* (Sms) and *Sistrurus m. miliarius* (Smm).

Protein Family	% of total venom proteins	
	Sms	Smm
Disintegrin	1.4	1.7
SVMPi	2.9	2.3
Vasoactive peptide	1.3	0.4
BPP	0.6	0.3
C-NP	0.8	<0.1
svNGF	0.7	–
CRISP	1.6	1.5
D49-PLA_2_	21.3	17.9
Serine proteinase	17.9	12.9
C-type lectin-like	1.8	–
SVMP	51.0	62.4
PI	1.1	0.7
PIII	49.9	61.7

### Phylogenetic Signal in Venom Composition and Diet

For all subsequent analysis we focused on 7 protein families (disintegrins, L-animo acid oxidase, CRISP, PLA2s, serine proteinases, SVMP, and PEP) each of which had abundances of >1% and were present in at least 6 of 8 taxa (Table 3). To generate composite variables for both venom and diet, we calculated PC 1 scores for both venom and diet and PC 2 for venom only and used these in subsequent analyses. For venom, PC 1 and 2 together account for 81% of the total variation in protein abundance (Table 4). The highest variable loadings for PC 1 are SVMP (0.92), PLA2 (−0.88), PEP (−0.42) and CRISP (0.41) whereas for PC 2 they are LAO (0.88), SP (0.72), CRISP (0.63), PLA2 (−0.45). For diet, PC 1 accounted for 87% of total variation and had a high negative loading for % mammals (−0.95) and positive loading for % lizards (0.91).

**Table 3:** Summary of proteomics and diet information used for comparative evolutionary analysis of rattlesnake venom composition.

	Protein family (% total venom proteins)												Diet	
Taxon	DISI	LAO	CRISP	PLA_2_	SP	SVMP	svNGF	CTL	MYO	DC	PEP	Kunitz	% mam	% liz
Sms	1.4	0	1.6	21.3	17.8	51.1	0.7	1.8	0	0	4.3	0	0.20	0.20
Smm	1.8	0	1.6	18.1	13.1	62.7	0	0	0	0	2.7	0	0.17	0.50
Smb	7.5	2.0	2.8	31.8	16.8	35.3	0.1	0.1	0	1.3	2.1	0.1	0.17	0.32
Sca	2.4	4.0	0.6	28.9	17.5	42.2	0.1	0.1	0.4	0.1	3.7	0	0.85	0.00
Sct	4.1	1.4	1.2	30.3	19.6	38.9	0.1	0.1	0.1	0.1	4.1	0	0.73	0.14
Sce	0.8	2.4	10.1	13.1	23.3	45.6	0.1	0.1	0	0.1	4.2	0.1	0.32	0.60
Catrox	6.2	8.0	4.3	7.3	19.8	49.8	0.0	1.6	0.0	0.0	3.0	0.0	0.87	0.05
Agc	1.3	0.4	3.7	31.7	14.3	32.5	0.3	2.4	0.0	0.0	0.7	0.0	0.37	0.06

Values represent % total venom proteome made up by a given protein as determined by HPLC analyses (see Methods). Abbreviations for names of protein families are as follows: DIS – Disintegrin; LAO – L-amino acid oxidase; CRISP – cysteine rich secretory protein; PLA_2_– phospholipase A_2_; SP – serine proteinase; SVMP – snake venom metalloproteinase; svNGF – snake venom nerve growth factor; CTL - C-type lectin-like; MYO – myotoxin; DC – Disintegrin-like/cysteine-rich SVMP C-terminal fragment; PEP - vasoactive peptides; Kunitz - Kunitz-type inhibitor. Diet measures: % mam – proportion of mammals in diet; % liz: proportion of lizards in diet. Taxon names: Sms – *Sistrurus m. streckerii*; Smm – *S. m. milarius*; Smb – *S. m. barbouri*; Sca – *S. c. catenatus*; Sct – *S. c. tergenimus*; Sce – *S. c. edwardsii*; Catrox – *Crotalus atrox*; Agc – *Agkstrodon c. contortrix*. Sources for proteomics and diet information are given in the Materials and Methods section.

**Table 4:** Measures of phylogenetic signal (estimated as K values) for venom and diet variation across the 8 taxa shown in Fig. 1.

Trait - venom	K-value	P-value
DISI	0.052	0.70
LAO	0.26	0.06
CRISP	0.04	0.83
PLA_2_	0.08	0.37
SP	0.07	0.50
SVMP	0.11	0.37
PEP	0.15	0.20
PC 1 - venom	0.09	0.37
PC 2 - venom	0.12	0.23
Trait - diet		
% mam	0.16	0.11
% liz	0.08	0.44
PC 1 - diet	0.11	0.22

P-value represents the probably that the observed value is greater then the expected value if trait values were randomly assigned to tip taxa. Trait names are as in Table 3 with the exception that PC 1 and 2– venom are the PC scores for composite venom variable and PC 1– diet are PC 1 scores for the composite diet variable. For venom, PC 1 and 2 explained 49% and 31% of total variation respectively, and had the following variable loadings (PC1 - DISI (−0.11); LAO (−0.36); CRISP (0.41); PLA2 (−0.88); SP (−0.12); SVMP (0.92); PEP (−0.42); PC2 - DISI (0.23); LAO (0.87); CRISP (0.64); PLA2 (−0.44); SP (0.72); SVMP (−0.33); PEP (0.12). For diet, PC 1 explained 87% of total variation and had the following loadings (% mammals (−0.95); % lizards (0.91).

Phylogeny-based comparative analysis of evolutionary change in venom compositon as well as diet show no evidence for a significant effect of phylogenetic signal in either set of variables. K values for individual venom components as well as PC 1 and 2 are mostly small (≤0.15) and none are significantly different from random (Table 4). The highest K value was observed for LAO (0.26) suggesting that of all the venom components analyzed it shows the strongest evidence for an assocation with phylogeny. However, this K value is also not signficant albeit marginally so (P = 0.06). Similar to venom, diet variation shows no association with phylogeny: K values for both % mammals and % lizards as well as PC 1 scores are also small (≤0.16) and not significantly different from random expectations. Overall both venom and diet are evolutionarily highly labile traits in these snakes.

### Associations between Diet and Venom Variation

The PGLS analyses allow us to look for evidence of correlated evolution between diet and venom composition independent of modest phylogenetic signal. Because % mammals and % lizards were inversely correlated with each other (r = −0.68) we focused on the diet PC 1 scores as overall measures of diet. Analyses based on % mammals and % lizards separately showed similar patterns to those based on diet PC 1 described below (results not shown). We initially found significant (P<0.05) correlations between diet PC 1 and CRISP (r = 0.26), PLA2 (−0.26) and venom PC1 scores (0.31) (Table 5); however, after adjusting the critical P-value for the number of tests performed, only the association between diet and CRISP protein variation remained significant, with snakes with high CRISP abundance having a greater abundance of lizards relative to mammals in their diets. Despite the small simple size, these analyses are consistent with a role for natural selection in molding variation in the abundance of some venom proteins in these snakes through variation in diet.

**Table 5:** Phylogenetic General Least Squares analyses for associations between diet and individual and composite venom traits for the 8 taxa shown in Fig. 1.

Trait - venom	Slope	P-value
DISI	−0.12	0.16
LAO	0.01	0.84
CRISP	0.26	0.003*
PLA_2_	−0.26	0.026
SP	0.03	0.50
SVMP	0.12	0.32
PEP	−0.01	0.67
PC 1 - venom	0.31	0.047
PC 2 - venom	0.18	0.12

Abbreviations for venom traits are given in Table 3. Diet was measured as composite trait PC 1 based on % mammals and % lizards in diet and has a high negative loading for % mammals and high positive loading for % lizards (see above).

* represents a p-value that remained significant after a FDR-based adjustment of critical p-value to 0.017 based on number of comparisons.

## Discussion

### Proteomic Analysis of Sistrurus Venom Composition

The proteomic characterization of *S. m. streckeri* and *S. m. miliarius* completes the venomic overview of genus *Sistrurus* initiated by Juárez *et al* [41] and Sanz *et al*. [23], and provides hints for understanding the toxinological profile and natural history of *Sistrurus* venoms.

The high abundance of extracellular matrix-disrupting Zn^2+^-dependent metalloproteinases (PIII-SVMP) [42], [43], cytolytic PLA_2_ molecules [44], thrombin-like serine proteinases affecting the blood coagulation cascade and platelet aggregation [3], and, to a lesser extent, hypotensive bradykinin-potentiating peptides (BPPs) [45]–[47] in both *S. miliarius* subspecies investigated suggests that the major effects of these venoms may involve local tissue damage, hemorrhage, coagulation disturbances, and vascular shock. Further, low abundant venom toxins such as disintegrins, C-type lectin-like molecules, and PI-SVMPs, which also interact with components of the human hemostatic system, may synergistically potentiate the activity of PIII-SVMPs and serine proteinases, resulting in increased incidence of bleeding and systemic disseminated coagulopathy (see discussion in Rodrigues *et al*. [48]).

Notwithstanding qualitative and quantitative differences, the large conservation (95%) in their venom proteomes underscores the close phylogenetic kinship of the two pigmy rattlesnake subspecies, which had a common ancestor as early as 0.49 Myr (Fig.1). In contrast, *S. m. streckeri* and *S. m. miliarius* share only a few venom proteins (disintegrin barbourin 4 [P22827], BPP/C-NP precursor 9 [B0VXV8], PLA_2_ 18 [ABY77929], NGF 14 [∼BAG16511], serine proteinase 23, PI-SVMP 29) with *S. m. barbouri*, indicating that only ∼24% of their venom proteomes has been preserved since their split 0.77 Mbp. These data highlight the evolutionary plasticity of pygmy rattlesnake venoms and also indicate two very different divergence rates across the *S. miliarius* clade: rapid differenciation of the venom proteomes of *Smb* and the common ancestor of *Sms* and *Smm* (∼9% divergence per 100,000 years) and a slow evolutionary rate (∼1% divergence per 100,000 years) for the venom proteomes of *S. m. streckeri* and *S. m. miliarius*. The high degree of differentiation among recently evolved subspecies points to a strong role for adaptive diversification via natural selection as a cause of this distinctiveness.

### Phylogentic Comparative Analyses of Venom Composition

Evidence for a strong association between venom composition and phylogenetic relationships of species comes from observations that broad-scale patterns of venom composition mirror deep phylogenetic relationships among venomous snakes (for review see [11]). For example, snakes in the family Elapidae tend to have smaller toxins such as three-finger toxins and PLA_2_s, whereas species in the family Viperidae have more high molecular weight toxins such as SVMPs. However, there are a number of examples where in more closely related, and well-defined lineages, patterns of venom composition do not closely reflect phylogenetic affinities. Specifically, rattlesnakes (*Crotalus* and *Sistrurus*), show variation in the presence of Type I (high levels of metalloprotease and low toxicity) versus Type II (low metalloprotease, high toxicity) venoms that shows no strong association with phylogeny [11].

Our analysis of the *Sisturus* venoms reported here is an example of the second pattern but with several differences. First, we compare venom composition among snakes that are exceptionally closely-related to each other phylogenetically, and probably represent a clade in the earliest stages of an adaptive radiation [22]. One implication is that, given the close relationships among the taxa analyzed, divergence effects should be especially strong and yet we observe little evidence of phylogenetic constraint operating on venom evolution, suggesting that phylogenetic effects are weak at best.

Second, our analyses are the first to apply phylogeny-based comparative methods to venom data generated using proteomics methods. These methods allow the characterization of venom composition to an unprecedented level of detail and as a result we are able to assess whether there is evidence for phylogenetic signal in the abundance of individual venom proteins across all taxa. As a result we are able to identify a large number of shifts in the composition of individual proteins that are statistically greater than expectations based on phylogenetic relationships among taxa. We found no evidence for a significant effect of signal on phylogeny-wide variation in any protein. In fact, K values for indivdual proteins are substantially lower than those observed for a wide range of traits in other species. Specifically, Blomberg et al [20] calculated K values for 119 traits that were associated with 34 different phylogenies from between six to 254 species. In contrast to our results, 92% of all traits in their analysis showed evidence for significant signal and mean K values for broad clases of traits (body size (mean K = 0.83), other morphological traits (0.70), life history traits (0.63), physiology (0.53), and behavior (0.35) were all greater than values observed for individual venom proteins and PC 1 and 2 scores). The small number of species in our analysis could have resulted in low statistical power in our ability to detect a significant K value [20] but the low observed K values demonstrate that even if they were significant the effects of phylogenetic divergence would be low. Overall, our result argues that the abundance of individual venom proteins are exceptionally evolutionarily labile traits and that phylogenetic divergence plays an extremely limited role in shifts in venom composition in terms of the abundance of specific types of proteins. This result leaves open the possiblity that another evolutionary force, possibly natural selection in relation to diet, plays a role in molding the venom composition of these snakes.

Our analysis makes a number of important assumptions. First, we use a single estimate of venom composition for each taxon based on a pooled sample to represent the venom profile of a taxon. In particular, samples from several taxa (e.g. *S. m. streckeri*) come from a single geographic location. This is of concern because geographic variation in venom composition certainly occurs in *Sistrurus* (for example, see 25). What is different between the proteomic data used in this study and other work in these snakes and others is that in general the venom variation is usually assessed at much more fine-scale (e.g. individual protein bands on 1-D PAGE gels) than was done here where we pooled venom proteins into broad categories of major protein families.

To assess whether there is significant geographic variation in venom composition characterized in this way, we reanalyzed proteomics-based venom profiles generated for individual snakes from three distinct geographic regions that span the western and central range of this species (Illinois, Ohio, and Ontario) as reported by Gibbs et al. [49]. For each snake, we generated values for % total venom made up by 5 major types of venom proteins (disintegrins, metalloprotenases, PLA2s, serine proteinases, and CRISP – for technical issues these were the only proteins that we were able to analyze) and compared proportions among individuals in each of the populations. The results show that only 2 of 15 population-by-protein comparisons were significant (p≤0.05) based on Mann-Whitney U tests. Our conclusion is that there is some possibility that geographic variation could be underestimated in our data but that this effect is limited, at least based on this analysis of data from this taxa.

We also assume that the relative percent composition made up by each venom protein has a genetic basis. Previous work has shown that venom composition based on HPLC profiles varies among individuals and that some of this variation is caused by differences in gene expression among individuals [26], [49]. We assume that the use of a pooled sample accounts for individual variation that may be present but the extent to which this is true is unknown. In terms of the genetic basis of protein variation, at least for one major protein family studied here (PLA_2_), Gibbs and Rossiter [50] showed a significant link between genomic and proteomic variation, supporting the assumption.

Finally an additional concern is that our use of proportions to measure the relative abundance of different proteins introduces a dependency among the values for individual proteins across taxa. Our transformation of the raw data using an arcsin square root transformation reduced this dependency but to an unknown level. This issue has been discussed for proportional data derived from HPLC analyses in chemical ecology and the use of a centered log ratio transformation has been proposed [51]. We chose not to use this transformation because of the difficulty of interpreting the meaning of parametric-based composite variables based on Principle Component analyses derived from centered log ratio transformed data. However, we suggest it would be fruitful to explore the use of this and possibly other transformations that minimize the covariation between indivdual values in future analyses.

### Causes of Venom Variation

Our results clearly demonstrate the both venom composition and diet are evolutionarily variable traits in these snakes which leaves open the possibility that adaptation by natural selection acting through diet variation could play a key role in molding venom variation. In support of a role for diet-mediated selection we found a strong positive correlation between CRISP abundance and snake diets that were high in lizards and low in mammals and weaker correlations between PLA2 abundance and a measure of overall venom composition in the form of PC 1 scores. These results focus attention on the CRISP venom protein as possibly playing a key role in mediating the effectiveness of rattlesnake venom in killing and/or digesting mammals and lizards and are consistent with recent molecular evolutionary analyses that found strong evidence for positive selection on the coding regions of CRISP venom genes in snakes [52]. Future work involving prey-specific toxicity tests which evaluated the effect of this protein and possibly others on mammals and lizards as described in [24] would be valuable in evaluating whether selection is a viable hypothesis to explain interspecific variation in these proteins.

Nevertheless, the fact that our independant contrast analyses failed to show correlations between diet and other major venom proteins which are major components of rattlesnake venom (e.g. snake venom metaloproteinases) is sobering. It is unclear whether this is due to shortcomings of our analyses in terms of the accuracy of our estimates of diet and venom composition of specific taxa, limited simple size or the primacy of other traits such as snake body size over venom in determining diet (see 32). A broad-based approach to developing evolutionary explanations for variation in major snake venom proteins should be an important focus of future studies of venom evolution in these rattlesnakes. However, our results make it clear that phylogenetic relatedness among species accounts for little of the observed variation and that causal explanations must involve other evolutionary and ecological mechanisms.

### Concluding Remarks

There has been a recent explosion of proteomic-based data on venom composition and this promises to continue in the future as the technological approaches become more refined and comprehensive genomic and/or transcriptomic databases become available [53]. At the same time, phylogenetic analysis of the relationships of the snakes producing these venoms has also increased dramatically [54], [55]. We feel that a joint analysis of these data sets using phylogenetic comparative methods like those described here offers an unprecedented opportunity to study venom evolution at a variety of time-scales to better understand the evolutionary forces that have shaped this variation in venomous snakes and will complement the handful of other studies that adopted the same general approach to studying the evolutionary basis for interspecific variation in venom composition [14], [56].

## Supporting Information

Figure S1 **Reverse-phase separation of Agkistrodon contortrix contortrix venom.** Chromatographic fractions were characterized by SDS-PAGE and assigned to protein families by de novo sequencing of in-gel trypsin digested protein bands (Table 1). Relative abundances of each protein family were computed from the sum of the relative chromatographic peak areas. SVMP, snake venom metalloproteinase; SP, serine proteinase; PEP, peptides; LAO, L-amino acid oxidase; DIS, disintegrin; CTL, C-type lectin-like; UNK, unknown; CRISP, cysteine-rich secretory protein; PLA2, phospholipase A2 (source: J. Calvete et al. unpublished ms.).
(TIF)

Table S1 **De novo assignment of RP-HPLC isolated fractions of Agkistrodon contortrix contortrix venom to protein families by MALDI-TOF-TOF or nESI-MS-MS (confidence ≥99%) of selected peptide ions from in-gel trypsin-digested protein bands.** Cysteine residues are carbamidomethylated; X: Leu/Ile; B: Lys/Gln. Confidence values were calculated by the Paragon algorithm of ProteinPilot® (source: J. Calvete et al. unpublished ms.).
(DOCX)
