# Supplementary figures and images for: Phylogeny-Based Comparative Analysis of Venom Proteome Variation in a Clade of Rattlesnakes (Sistrurus sp.)
Source: PLoS One. 2013 Jun 24;8(6):e67220. doi: 10.1371/journal.pone.0067220 (PMC3691181; doi:10.1371/journal.pone.0067220)

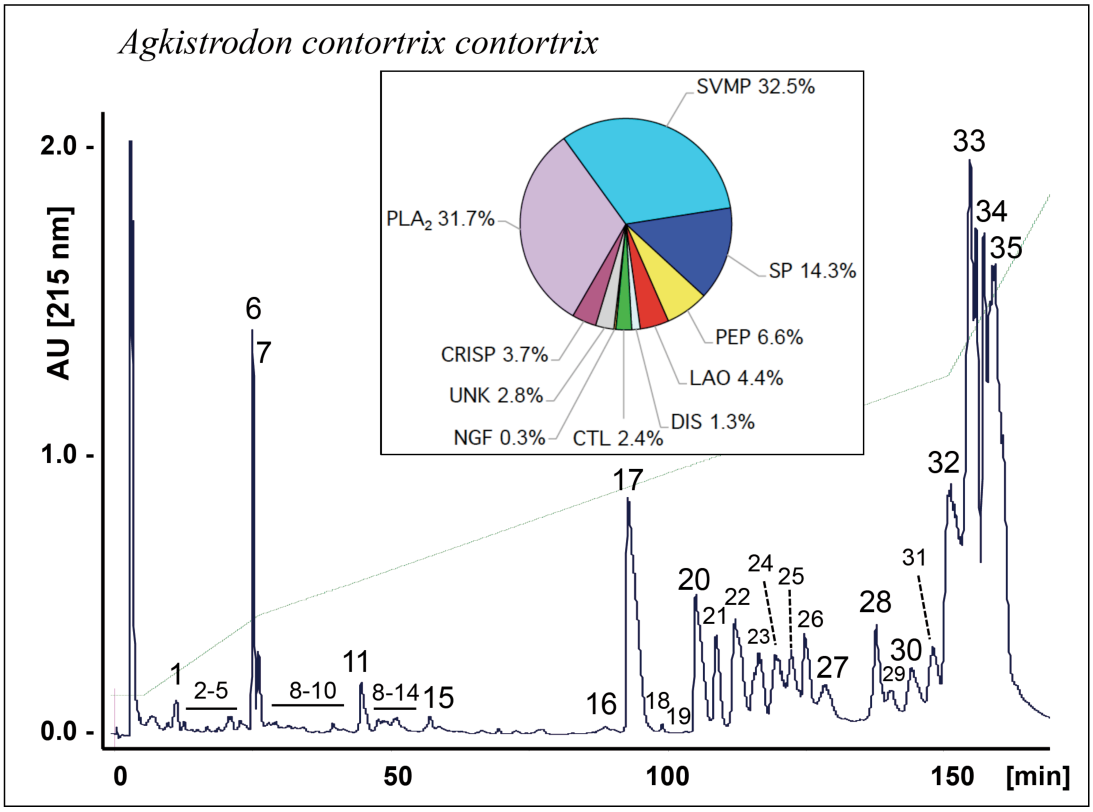

Supplement: Figure S1 — Reverse-phase separation of Agkistrodon contortrix contortrix venom. Chromatographic fractions were characterized by SDS-PAGE and assigned to protein families by de novo sequencing of in-gel trypsin digested protein bands (Table 1). Relative abundances of each protein family were computed from the sum of the relative chromatographic peak areas. SVMP, snake venom metalloproteinase; SP, serine proteinase; PEP, peptides; LAO, L-amino acid oxidase; DIS, disintegrin; CTL, C-type lectin-like; UNK, unknown; CRISP, cysteine-rich secretory protein; PLA2, phospholipase A2 (source: J. Calvete et al. unpublished ms.). (TIF) [file pone.0067220.s001.tif]
