# Supplementary material for: Phylogeny-Based Comparative Analysis of Venom Proteome Variation in a Clade of Rattlesnakes (Sistrurus sp.)
Source: PLoS One. 2013 Jun 24;8(6):e67220. doi: 10.1371/journal.pone.0067220 (PMC3691181; doi:10.1371/journal.pone.0067220)
Supplement: Table S1 — De novo assignment of RP-HPLC isolated fractions of Agkistrodon contortrix contortrix venom to protein families by MALDI-TOF-TOF or nESI-MS-MS (confidence ≥99%) of selected peptide ions from in-gel trypsin-digested protein bands. Cysteine residues are carbamidomethylated; X: Leu/Ile; B: Lys/Gln. Confidence values were calculated by the Paragon algorithm of ProteinPilot® (source: J. Calvete et al. unpublished ms.). (DOCX) [file pone.0067220.s002.docx]

**Table S1**: *De novo* assignment of RP-HPLC isolated fractions of *Agkistrodon contortrix contortrix* venom to protein families by MALDI-TOF-TOF or nESI-MS-MS (confidence ≥ 99%) of selected peptide ions from in-gel trypsin-digested protein bands. Cysteine residues are carbamidomethylated; X: Leu/Ile; B: Lys/Gln. Confidence values were calculated by the Paragon algorithm of ProteinPilot^®^.

| **HPLC peak** | **Mass (kDa)** | **Peptide Ion** | | **MS/MS-derived or N-terminal sequence** | **Protein family** | **~ related protein** |
| --- | --- | --- | --- | --- | --- | --- |
|  |  | **m/z** | **z** |  |  |  |
|  |  |  |  |  |  |  |
| 1 |  | 532.3 | 2 | TPPAGPDVGPR | BIP | P85025 |
| 2-5 |  | 865.4 | 1 | PAGPDVGPR | BIP | P85025 |
|  |  | 481.7 | 2 | PPAGPDVGPR | BIP | P85025 |
|  |  | 592.2 | 2 | TPNLTPEQQR | N-terminal fragment PII-SVMP | ACJ61245 |
|  |  | 398.8 | 2 | KVTPVPR | Fragment PI-SVMP | ACV83930 |
| 6 |  | 430.1 | 1 | ZNW | SVMPi | ~P01021 |
| 7 |  | 444.4 | 1 | ZBW | SVMPi | ~P01021 |
| 8-10 |  | 1967.8 | 1 | LTPGAQCAEGLCCDQCK | Disintegrin | ~ C9E1S2 |
| 11 | 11 | 1870.8 | 1 | GDDVNDYCNGISAGCPR | Disintegrin | ~ C9E1S2 |
|  |  | 1967.8 | 1 | LTPGAQCAEGLCCDQCK |  |  |
| 12-15 | 13 | 2059.9 | 1 | NAITSYGSYGCNCGWGHR | K_49_-phospholipase A_2_ | ~ P49121 |
| 16 | 24 | 1111.6 | 1 | NPNPVPTGCR | Nerve growth factor | ~ Q9DEZ9 |
| 17 | 13 | 2059.9 | 1 | NAITSYGSYGCNCGWGHR | K_49_-phospholipase A_2_ | ~ P49121 |
| 18 | 17 | 1505.6 | 1 | CCFVHDCCYGK | K_49_-phospholipase A_2_ | ~ Q91506 |
| 19 | 31 | 1186.8 | 1 | FLVALYTFR | Serine proteinase | ~ P0CV90 |
| 20 | 30 | 1597.8 | 1 | CANINILDYSVCR | Serine proteinase | ~ Q9DF68 |
|  | 24 | 1537.7 | 1 | MEWYPEAAANAER | CRISP | ~ Q7ZTA0 |
|  |  | 1195.6 | 1 | SVDFDSESPR |  |  |
| 21 | 12 | 1206.6 | 1 | LWNDQVCGSK | C-type lectin-like | ~ Q9PSN0 |
|  |  | 1401.7 | 1 | GQAEVWIGLWDK |  |  |
| 22 | 32 | 2562.3 | 1 | TFLCGGTLINQEWVLTAAHCDR | Serine proteinase | ~ P09872 |
|  | 29 | 2856.4 | 1 | GDSGGPLICNGQFQGILSVGGNPCAQPR | Serine proteinase | ~ P09872 |
|  | 26 | 1097.7 | 1 | FLALVYTDR | Serine proteinase | ~ Q072L6 |
| 23 | 26 | 2119.2 | 1 | SAHIAPLSLPSSPPSVGSVCR | Serine proteinase | ~ Q9YGJ9 |
|  | 24 | 2206.1 | 1 | LLDDAACQPGYPEVLPEYR | Serine proteinase | ~ P82981 |
| 24,25 | 25 | 1498.7 | 1 | VVGGDECNINEHR | Serine proteinase |  |
| 26 | 27-37 | 1639.8 | 1 | CANINLLDYEVCR | Serine proteinase |  |
| 27-29 | 49 | 1513.7 | 1 | ETNYEEFLEIAR | L-amino acid oxidases | ~ Q6STF1 |
| 28,29 | 42 | 1803.7 | 1 | YFVEVGEECDCGSPR | PIII-SVMP | ~ Q9DGB9 |
|  |  | 1684.7 | 1 | GAQCAEGLCCDQCR |  |  |
| 30 | 46 | 1442.6 | 1 | DECDMADLCTGR | PIII-SVMP | ~ Q9DGB9 |
| 31 | 72 | 1983.9 | 1 | LTPGSQCADGVCCDQCR | PIII-SVMP | ~ O42138 |
|  | 62 | 1699.9 | 1 | NQCIYFFGPNAAVAK | PIII-SVMP | ~ O42138 |
|  |  | 1734.7 | 1 | MYDIVNVITPIYHR |  |  |
| 32 | 26 | 1187.6 | 1 | TPEQQGFPQR | PI-SVMP | ~ Q92031 |
|  |  | 3122.6 | 1 | HDNAQLLTAIDFDGDTVGLAYVGGMCQLK |  |  |
| 33 | 23 | 2564.4 | 1 | ISHDNAQLLTAIELDGETIGLANR | PI-SVMP | ~ P28891 |
| 34 | 26 | 1313.8 | 1 | YVELVIVADHR | PI-SVMP | ~ Q92031 |
|  |  | 2169.2 | 1 | SHDNAQLLTAIVFDEGIIGR | PI-SVMP | ~ B7U492 |
| 35 | 23 | 1327.87 | 1 | YVELVIIADHR | PI-SVMP | ~ B7U492 |
|  |  | 1201.6 | 1 | APLAGMCDPNR |  |  |
